# Unveiling the microwave heating performance of biochar as microwave absorber for microwave-assisted pyrolysis technology

**DOI:** 10.1038/s41598-024-59738-5

**Published:** 2024-04-22

**Authors:** Rickwinder Singh, Christoph Lindenberger, Aakash Chawade, Vivekanand Vivekanand

**Affiliations:** 1 Centre for Energy and Environment, Malaviya National Institute of Technology Jaipur, Jaipur, Rajasthan 302017 India; 2 University of Applied Sciences Amberg-Weiden, Kaiser-Wilhelm-Ring 23, 92224 Amberg, Germany; 3 Department of Plant Breeding, Swedish University of Agricultural Sciences, 23053 Alnarp, Sweden

**Keywords:** Environmental sciences, Energy science and technology

## Abstract

Microwave (MW) heating has gained significant attention in food industries and biomass-to-biofuels through pyrolysis over conventional heating. However, constraints for promoting MW heating related to the use of different MW absorbers are still a major concern that needs to be investigated. The present study was conducted to explore the MW heating performance of biochar as a low-cost MW absorber for performing pyrolysis. Experiments were performed on biochar under different biochar dosing (25 g, 37.5 g, 50 g), MW power (400 W, 700 W, 1000 W), and particle sizes (6 mm, 8 mm, 10 mm). Results showed that MW power and biochar dosing significantly impacted average heating rate (AHR) from 17.5 to 65.4 °C/min at 400 W and 1000 W at 50 g. AHR first increased, and then no significant changes were obtained, from 37.5 to 50 g. AHR was examined by full factorial design, with 94.6% fitting actual data with predicted data. The model suggested that the particle size of biochar influenced less on AHR. Furthermore, microwave absorption efficiency and biochar weight loss were investigated, and microwave absorption efficiency decreased as MW power increased, which means 17.16% of microwave absorption efficiency was achieved at 400 W rather than 700 W and 1000 W. Biochar weight loss estimated by employing mass-balance analysis, 2–10.4% change in biochar weight loss was obtained owing to higher heating rates at higher powers and biochar dosing.

## Introduction

Improper waste management and dependency on fossil fuels are still global concerns contributing to severe socioeconomic and environmental challenges^1,2^. Several nations have implemented strict regulations for waste collection, segregation, and disposal. However, these wastes have great potential along with other biomasses for their utilization in bioenergy production by employing various techniques^3,4^. As the employment of bioenergy in different energy production systems has been augmented by an average of 3%/year in the duration of 2010–22. As per the IEA report, the use of bioenergy is expected to increase approx. 8% by 2030 which will be the stagnant approach for waste utilization and emissions mitigation^5^. Furthermore, significant efforts are required to employ advanced technologies for modern bioenergy production, which will help promote the concept of net zero emission and circular bioeconomy^6^. For the conversion of waste to bioenergy, pyrolysis technology is a promising thermochemical process that can be employed to transform any organic waste into valuable products, mainly biofuels^7,8^. In the past decade, researchers have used conventional pyrolysis, which works on the principle of conduction, convection, and radiation, to treat feedstocks for their conversion to biofuels. However, some challenges are related to low energy efficiency, poor quality of biofuels and drastic heat loss^9^. To overcome these challenges related to conventional pyrolysis, MW-assisted pyrolysis has received a lot of attention owing to unique heating/volumetric heating (dielectric heating^10^) and efficient methods. In this technology, MW radiation acts directly to the core of feedstock that MW energy converts into heat from the inner core to surface of feedstock owing to particle collusions via dipole polarization, ion conduction, and interfacial polarization, providing high energy utilization efficiency^11^. Therefore, this type of heating provides selective heating, higher heating rates which help to improve quality products and pyrolysis efficiency. Dielectric heating requires the materials to have higher MW absorbing or dielectric properties to reach pyrolysis temperature. However, biomasses and organic waste have low susceptibility towards MW irradiation and dielectric properties, which hinders to achieving the desired pyrolysis temperature and results in low energy utilization efficiency^11,13^.

Aguilera et al.^14^ developed a kinetic model for a MW reactor for aqueous semi-liquid material for epoxidation. Further, the effect of MW radiation on materials was analyzed in which polar molecules acted as positively and negatively charged depending on MW irradiation impact. Due to this, MW resonance^15^ is produced, which helps to increase the temperature of materials under MW action. This MW resonance may depend on different materials and their polarity as well as dielectric properties^16^. Thus, it becomes necessary to study and analyze heating performance as well as other parameters such as type of absorber, particle size, effect of MW power and amount of absorber^17^. Researchers have employed different MW absorbers and investigated their influence on pyrolysis and product quality. Suriapparao et al.^18^ have examined the effect of MW power and MW absorber quantity (graphite) on MW pyrolysis of waste polypropylene. They have achieved a maximum heating rate of ~ 71.4 °C/min at a higher amount of graphite and 600 W for the production of higher bio-oil yield. Furthermore, Zhou et al.^19^ have investigated the step toward scaling up MW pyrolysis for syngas production from sludge. Researchers have observed that sludge has low susceptibility to MW heating; thus, silicon carbide (SiC) has been employed to reach the desired temperature of 800 °C for pyrolysis. However, the use of SiC has some disadvantages related to its cost, large amount required for upscaling, and formation of amalgamation with by-products of pyrolysis process. In another study, Hou et al.^20^ have employed the steel slag catalyst (SSC) containing Fe_2_O_3_, Fe_3_O_4_, and MnFe_2_O_4_ as an absorber in MW pyrolysis for recovery of bioenergy from waste engine oil by converting into bio-oil. In this study, SSC was activated by alkaline and acidic pretreatments, which provided a maximum temperature of ~ 650 °C in 15–20 min under MW irradiation for pyrolysis.

It was reported that MW absorbers have great significance in operating and upscaling MW pyrolysis technology which motivates to analyze the selection, heating performance and energy absorption efficiency of MW absorbers. Wang et al.^21^ have examined the microwave absorption efficiency of water, glycerol, paraffin oil; and analyzed the various parameters such as MW power (MWP), feed & furnace size. Results showed that these parameters affected microwave absorption efficiency and MW heating performance. Further, particle size of absorbers has been optimized around 10 mm by Zhang et al.^22^ which can efficiently provide better microwave absorption efficiency. From these studies, it testified that the effect of MW heating can be improved by employing MW absorbers for various applications such as heating and thermochemical processes, mainly torrefaction and pyrolysis. However, it is required to optimize various critical factors like the design of MW setup, MW power, type, particle size and amount of absorber which primarily affect the weight loss under MW irradiation, heating performance and microwave absorption efficiency of absorbers^23,24^.

Several MW absorbers have been employed in MW heating for thermochemical processes. Among all, SiC is the most prominent contenders which have significant properties such as heating performance, dielectric property, good stability at higher temperatures. Ke et al.^25^ have investigated the heating performance and microwave absorption efficiency of different SiC amounts (20 g-40 g) under MW heating, which means different MW power (300–500 W). It observed that average heating rate obtained as 7.08 °C/s, whereas microwave absorption efficiency was increased from 4.7 to 37.38% at MW power of 300–500 W. It showed that heating rate depends upon the increasing the MW power and SiC dosing. However, SiC provided very high heating rate whereas it may form amalgamation with biochar or carbon residues in MW pyrolysis. Moreover, it is difficult to separate from carbon residues after pyrolysis which is still problem related to SiC as MW absorber. Beside SiC, biochar/charcoal can be another promising MW absorber which have several advantages such as low cost, no need to separate from carbon residues from pyrolysis, ease of reusability, etc. over other MW absorbers. As Fricler et al.^26^ investigated the effect of different MW absorbers (bentonite, chalk, eggshell, carbon residue/biochar and charcoal) on the production of syngas from MW pyrolysis of agricultural residues. Results showed that carbon residues or biochar from waste materials provided better quality and maximum syngas at 700 W MW power. Thus, it is necessary to examine heating performance and microwave absorption efficiency of biochar/carbon residues under action of MW heating, which is the novelty of this proposed work. To best of author's knowledge, no study has been reported on analysis of the heating performance of biochar/carbon residues as a MW absorber under action of MW heating for thermochemical processes.

This study aims to investigate biochar's heating performance and microwave absorption efficiency as MW absorber under the action of MW heating. Furthermore, the effect of factors such as MW power, particle size, and absorber dosing on the heating performance of biochar have been analyzed by using the design of experiments. After that, weight loss and changes in the properties of absorber were investigated after MW heating. This study will dominate the use of biochar as a MW absorber for performing various thermochemical processes mainly pyrolysis, and torrefaction under MW heating.

## Results and discussion

In this section, the findings of this study have been presented, initially, the effects of factors on heating performance of biochar have been analyzed. Further, the temperature profile of biochar heating under different conditions mainly varies between MW irradiation/power, biochar dosing, and biochar particle size.

### Temperature profiles of biochar heating under different conditions

**Figure 1:**
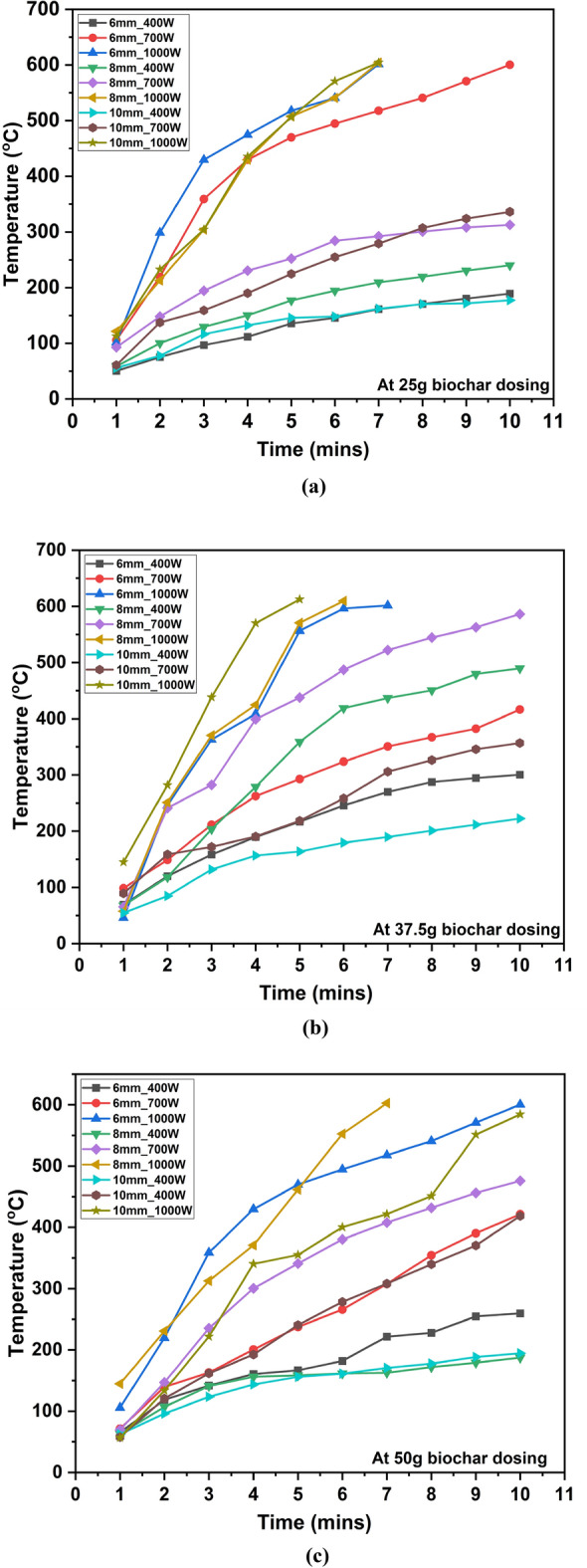
The temperature profiles under MW heating (400 W, 700 W, 1000 W) of biochar at dosing varied (**a**) 25 g, (**b**) 37.5 g, (**c**) 50 g respectively.

Figure 1a–c shows the temperature profile of biochar under MW heating at different hardwood biochar dosing, MW power levels, and particle sizes. It is found that temperatures of biochar have increased for all biochar dosing at various MW powers. Further, it was followed by a slow rise in temperature due to higher MW absorbability at lower temperatures than higher temperatures. This trend may vary for different biochar types produced from various feedstocks. From the experimental work, maximum temperature (~ 600 °C) was achieved within only 7 min at 1000 W at 25 g of biochar dosing for all particle size as shown in Fig. 1a; this can be attributed to higher MW power leads to rapid temperature rise. The initial rapid temperature rise was seen in case of 6 mm particle size, 25 g biochar dosing and 1000 W, which may be attributed to low particle size/higher surface area than other particle sizes (8 mm & 10 mm) leading to higher MW irradiation absorption^25,27^. For other MW powers (400 W and 700 W), there was an irregular temperature profile showing that a large amount of MW heat dissipated owing to a small sample size (25 g). On other side, lower MW power (400 W) facilitated to reach only 210 °C within 10 min owing to lower MW absorbance of biochar at 400 W. Although maximum temperature rise (~ 600 °C) was obtained earlier within in 5 min as biochar dosing increased from 25 to 37.5 g at 1000 W and particle size of 10 mm. Also, slightly different temperature rise was found as particle size of hardwood biochar varied from 6 to 8 mm in case of 37.5 g biochar dosing rather than others. Also, it means that the particle size of biochar used has slightly affected the temperature profile under MW heating. Whereas some uneven changes were assessed in case of 50 g of biochar dosing at all MW powers. It was detected that the medium temperature rose initially at 50 g biochar dosing, and after that, it slowly increased, which showed better results than 25 g dosing. However, it provided a slightly fragile temperature profile owing to uneven variations in temperature rise because the higher amount of biochar dosing (50 g) may be required a lot of MW energy to reach higher temperatures. Overall, these variations showed that MW heating is unable to provide uniform heating initially due to variable properties of MW absorber which was also analyzed for biochar under MW heating^28^. MW heating has excellent potential to treat the biomass/organic waste to produce valuable products with different thermochemical processes (hydrothermal carbonization, Torrefaction, Pyrolysis) at required temperatures (180–240 °C, 200–300 °C and 300–600 °C) respectively^29,30^.

### Analysis of factors affecting the heating performance of biochar

A polynomial model considering the quadratic polynomial function of 3 factors with multilevel was employed to analyze the effect of various factors considered in this study. This model to predict heating performance is examined by a quadratic model of followed the Eq. (1) form:

1 $${\text{Y}} =\upbeta _{0} +\upbeta _{1} {\text{X}}_{1} +\upbeta _{2} {\text{X}}_{2} +\upbeta _{3} {\text{X}}_{3} +\upbeta _{12} {\text{X}}_{1} {\text{X}}_{2} +\upbeta _{13} {\text{X}}_{1} {\text{X}}_{3} +\upbeta _{23} {\text{X}}_{2} {\text{X}}_{3}$$

where Y is the response (AHR), β_0_ is the constant regression coefficient, β_i_ and β_ii_ are the linear and quadratic regression coefficients, β_ij_ (i, j = 1,2,3) are the regression coefficients of 2-factor interactions, respectively. The regression coefficient (β) represents the typical increase in dependent variable when the independent variable increases by one unit, while keeping other independent variables constant. Also, X_1_, X_2_ and X_3_ represents the parameters such as Biochar dosing (g), Particle size (mm), MW power (W) respectively. Further, linear regression and analysis of variance (ANOVA) were employed to check the effect of each factor individually and their interactions. The results of ANOVA test were given in Table 1.

**Table 1:** ANOVA for full factorial model.

Source	Sum of squares	dof	Mean square	F-value	*p*-value	Significance
Model	5390.61	18	299.48	28.34	< 0.0001	High
A-Biochar dosing	318.43	2	159.21	15.07	< 0.0019	Medium
B-Particle size	6.24	2	3.12	0.2950	0.7523	None
C-MW power	4851.73	2	2425.87	229.56	< 0.0001	High
AB	52.19	4	13.05	1.23	0.3693	None
AC	115.35	4	28.84	2.73	0.1059	Medium
BC	46.68	4	11.67	1.10	0.4174	None
Residual	84.54	8	10.57	–	–	–
R^2^	0.9846 (98.46%)					
Adjusted R^2^	0.9498 (94.98%)					

**Figure 2:**
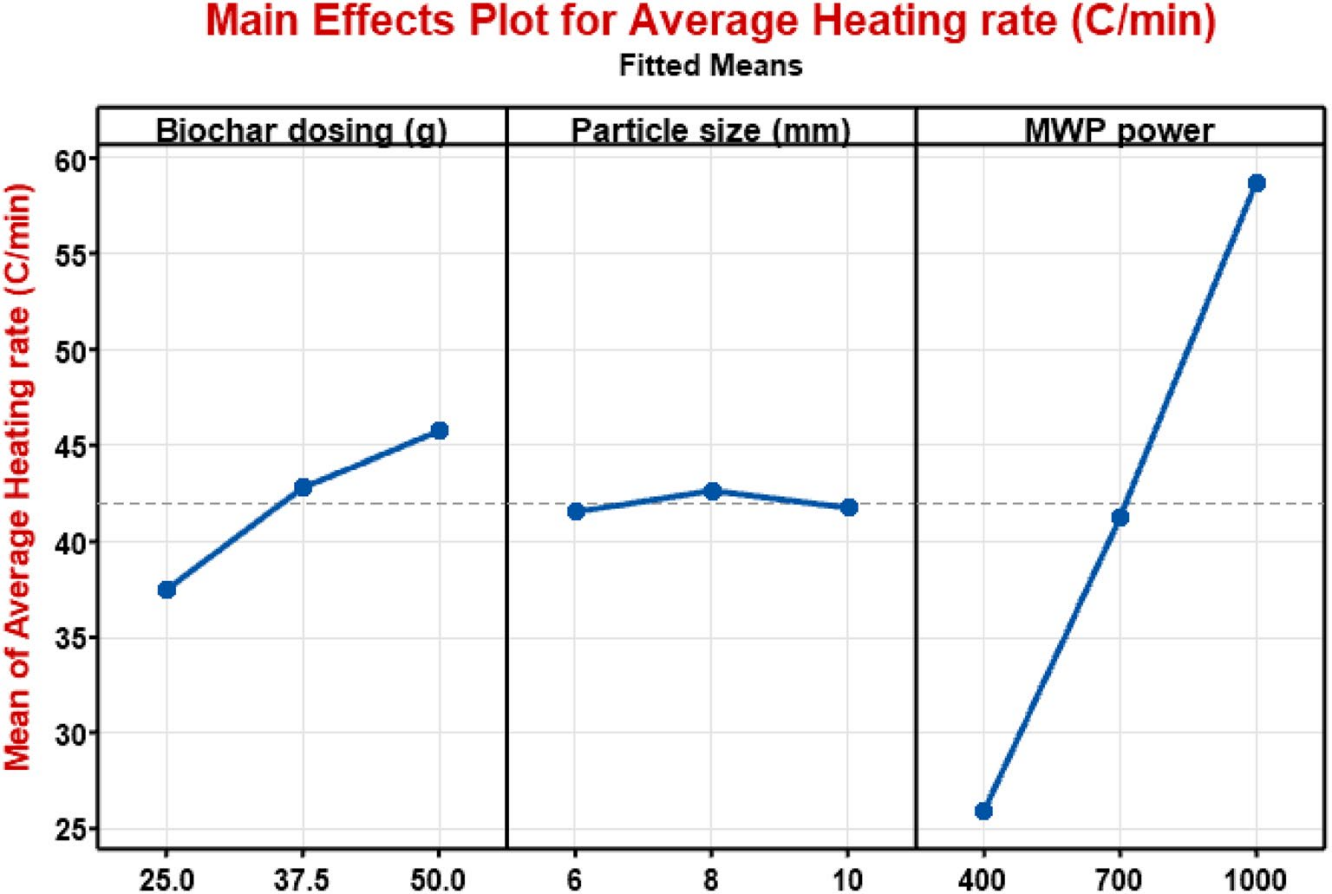
Main effects of biochar dosing, particle size and MW power on average heating rate.

**Figure 3:**
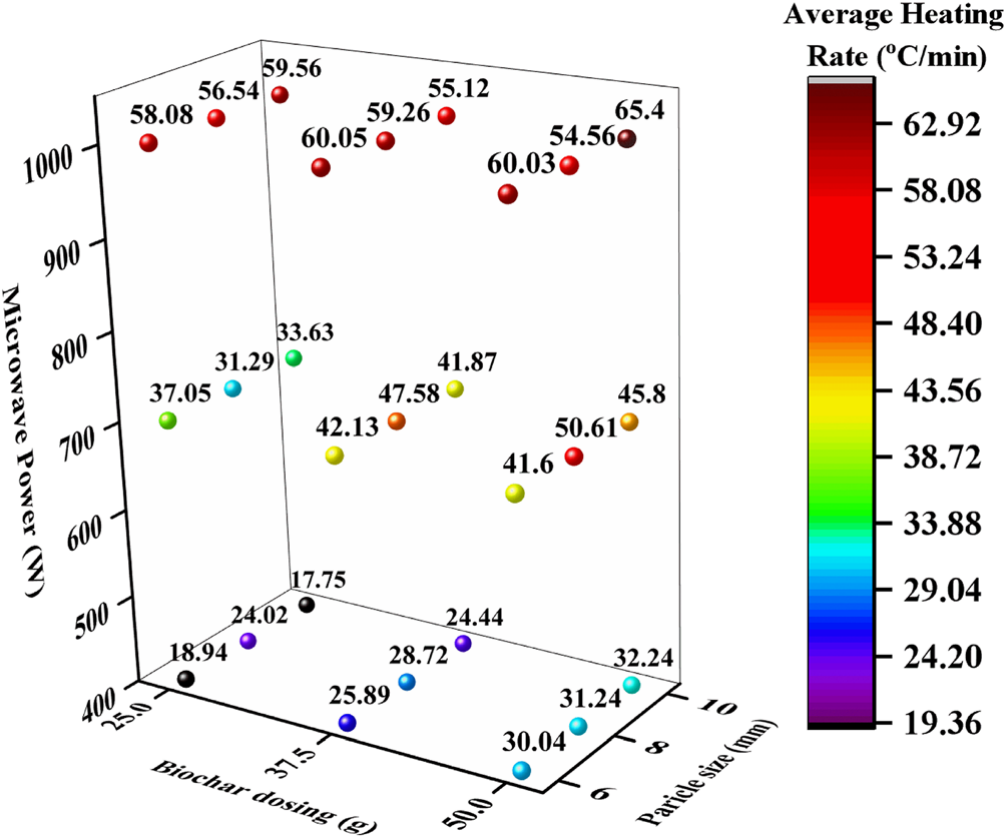
Depiction of average heating rate (°C/min) at different operating parameters (MW power, biochar dosing and particle size) have been plotted.

The effects of biochar dosing, particle size, and MW power on the heating performance, mainly AHR are assessed by employing full factorial design. Figure 2 depicts the main effects of process parameters on AHR (°C/min). It found that main effect of biochar dosing has medium level effect on AHR. As biochar dosing increased from 25 to 50 g, 27.1% heating rate increased under MW heating owing to higher amount of biochar provided maximum number of hot spots compared to lower amount of biochar^22,31^. Further, another parameter particle size (mm) had little effect on AHR, this may be attributed due to the porosity and surface area of used biochar closely varying means biochar produced from single biomass (hardwood). It is found that among all particle size of biochar, 8 mm particle size provided significant results as compared to 6 mm and 10 mm means biochar particle size of ~ 8 mm can be used to further perform research studies of MW heating for thermochemical conversion processes. Apart from particle size and biochar dosing, MW power greatly affected the AHR compared to particle size and biochar dosing. Results of AHR significantly affected by MW power as increasing as 400 W, 700 W and 1000 W. Figure 3 shows that AHR was evaluated by performing the experiments at different combinations of operating conditions such as MW power, biochar dosing, particle size. It was observed that AHR increased as MW power and biochar dosing were increased. However, there is slight change acquired by varying the particle size simultaneous to other variables. In Fig. 3, results showed the maximum average heating rate achieved as 60.03–65.4 (°C/min) at 1000 W and 50 g biochar dosing due to increased MW irradiation and higher amount of biochar absorbed maximum MW energy, respectively.

Furthermore, increasing the biochar dosing from 25 to 50 g provided the increment of 58.6% in AHR as follows 18.94 (°C/min) to 32.24 (°C/min) at 400 W and ranging 6–10 mm particle size which means that this combination can be further applied for mild/slow pyrolysis or torrefaction of various biomasses to producing maximum yield of carbon residues. These carbon residues can be used to capture carbon which may be used for several applications such as carbon fuel, soil conditioner, for treatment of wastewater, building material and many others. Moreover, biochar dosing in MW heating can provide a significant amount of energy at 700 W and 1000 W, as 19.86% and 61.76% of increments in heating rates were obtained, respectively. The average values of heating rates obtained were in the range of 17.75 to 32.24, 31.29 to 50.61 °C/min, °C/min and 54.56 °C/min to 65.4 °C/min at 400 W, 700 W and 1000 W respectively corresponding to different biochar dosing and particle sizes as shown in Fig. 3.

**Figure 4:**
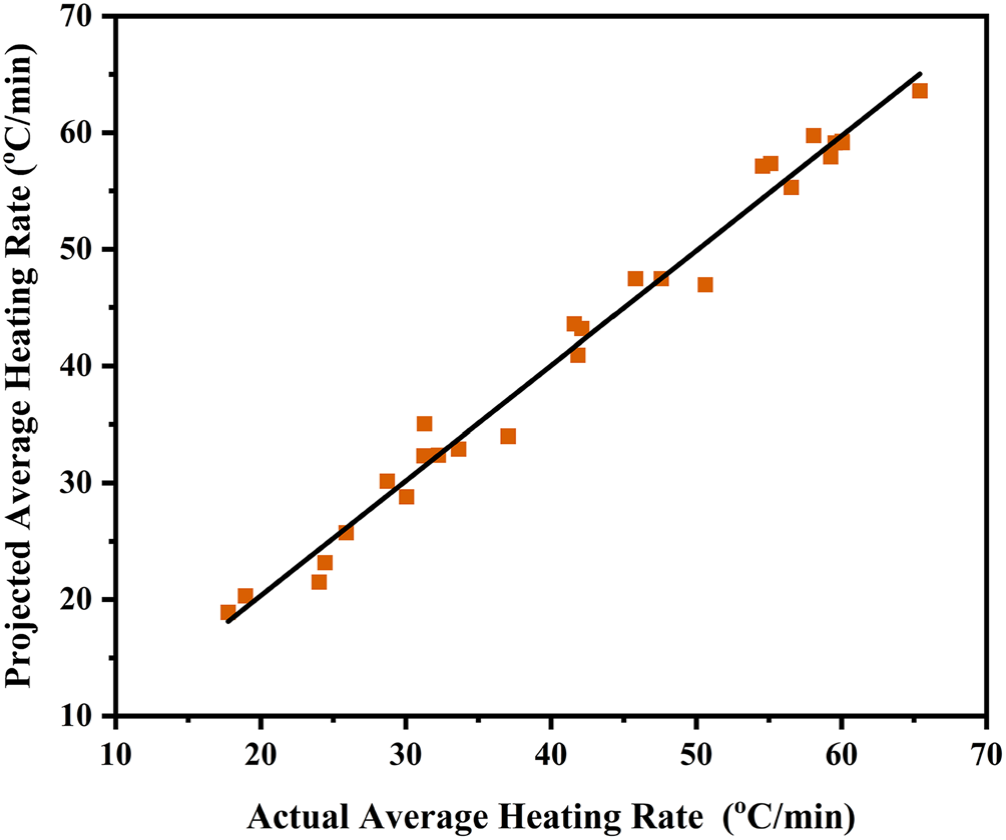
The Predicted versus Actual data of average heating rate by using regression model was analyzed which shows that actual data fits well ~ 94.98% with predicted data.

This type of combination can be employed biochar as MW absorber in pyrolysis further for production of bio-oil and syngas from biomass and organic waste. Furthermore, it is required to check whether model is fit or lack of fit which was analyzed by comparing the actual data of average heating rate (°C/min) with predicted data obtained from linear regression modelling as shown in Fig. 4. From full factorial design and linear regression analysis, predicted values of model fitted R^2^ =  ~ 94.98% with experimental results of average heating rate and provided the significance of chosen factors.

### Analysis of MW absorption efficiency and biochar weight loss

To examine MW heating performance, it is required to analyze the efficiency of this type of heating and degradation of MW absorber. This section examines the MW absorption efficiency and biochar weight loss under MW heating at different conditions.

#### Microwave absorption efficiency

**Figure 5:**
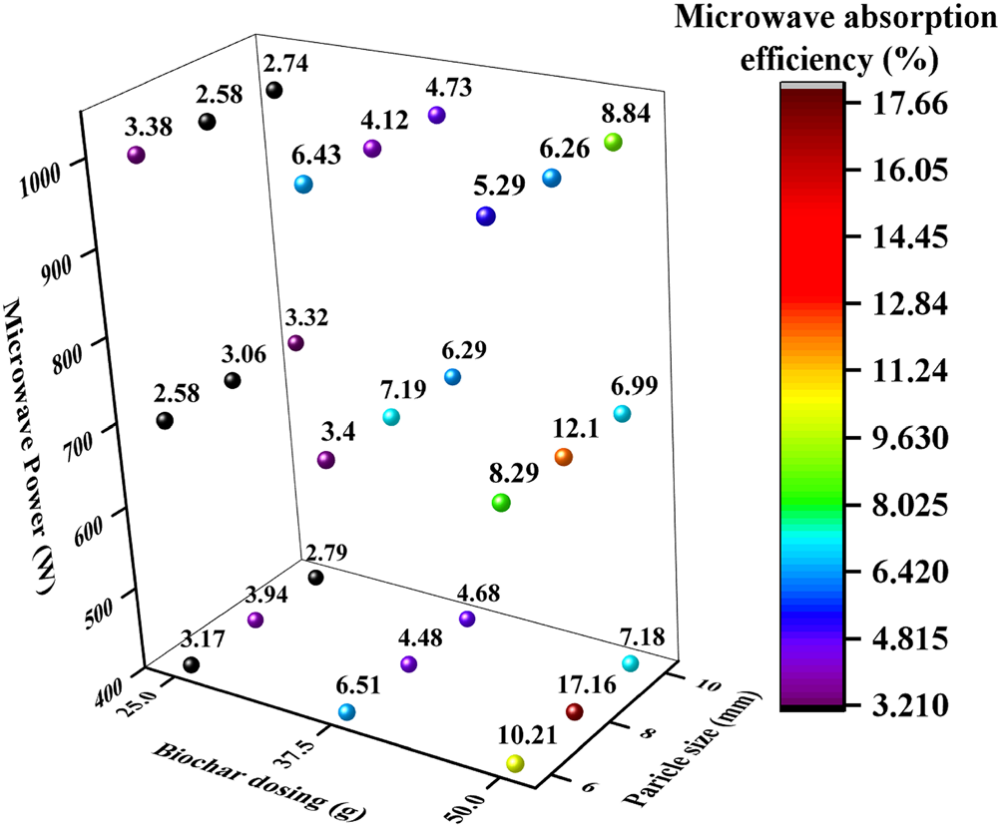
The variation in MW absorption efficiency (%) at different parametric conditions under MW heating.

Figure 5 shows the microwave absorption efficiency of hardwood biochar at different parametric values under MW heating. As per MW absorption theory, three phenomena can occur: reflection, absorption and transmittance during MW heating^32^. In this study, MW absorption was analyzed, considering other phenomena, mainly transmittance and reflection, as heat losses during experimentation. The analysis of results depicted in Fig. 5 showed a decrease in microwave absorption efficiency examined as MW power increased from 400 to 1000 W owing to a lot of MW loss at higher power. This may be attributed to less sample weight of biochar dosing (25 g, 37.5 g and 50 g) means maximum capacity of samples may be reached at lab scale. It would be increased when it will use at pilot and industrial scale. Furthermore, biochar dosing also affected the microwave absorption efficiency by increasing the biochar dosing (25–50 g) led to microwave absorption efficiency because higher dosing absorbed maximum MW irradiation. At 400 W, microwave absorption efficiency obtained as follows 3.17% to 10.21%, 3.94% to 17.16% and 2.79% to 7.18% at different values of biochar dosing (25 g, 37.5 g and 50 g) and particle sizes (6 mm, 8 mm, 10 mm). However, decreasing trend has been seen in the case of 700 W and 1000 W as compared to 400 W owing to low power and slow heating rates. On other side, particle sizes (6 mm, 8 mm, 10 mm) also influenced on microwave absorption efficiency, among all 8 mm of particle size of biochar provided significant/maximum increase in microwave absorption efficiency ranging 6.26%, 12.1% and 17.16% at different MW powers (400 W, 700W  and 1000 W) at 50 g of biochar dosing. Overall, microwave absorption efficiency decreased as MW power increased and 50 g and 8 mm provided higher microwave absorption efficiency at all MW power.

### Weight loss of biochar and its reusability

**Figure 6:**
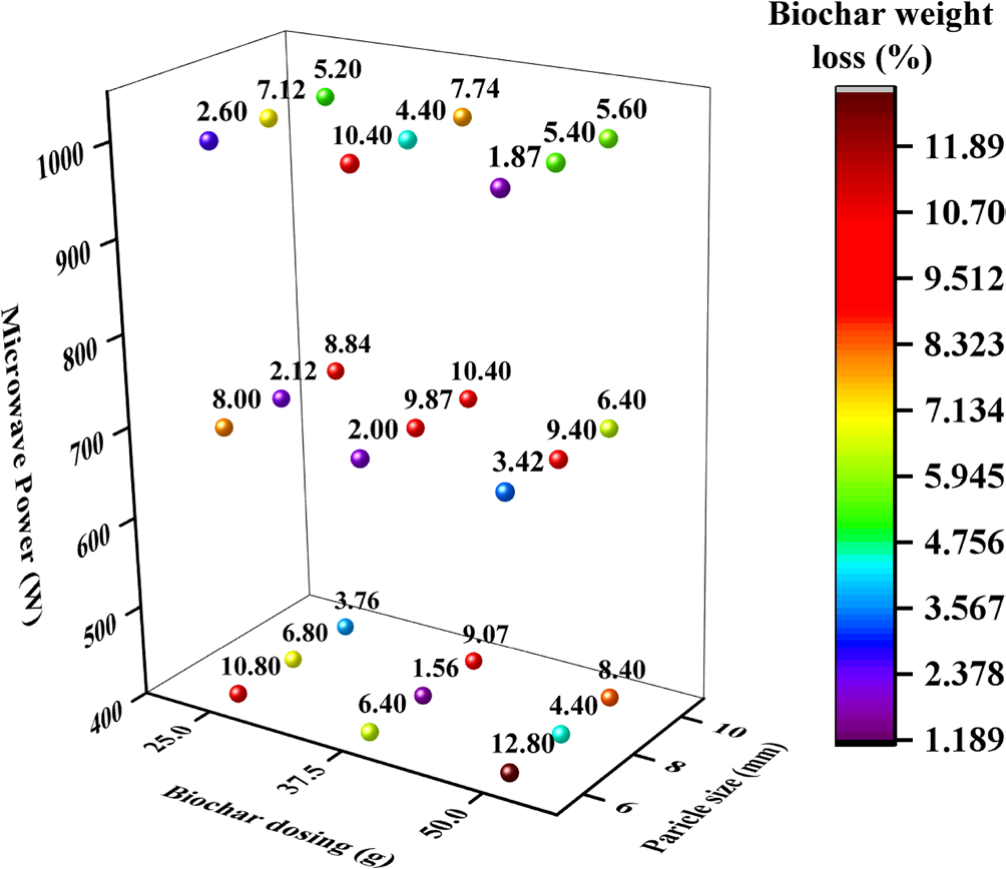
Explores the estimation of biochar weight loss under MW heating.

Figure 6 explains the biochar/MW absorber wright loss under MW heating during the experimentation of this study. Since it is required to analyze the degradation of the absorber which is being used to perform MW heating processes depending upon the requirements so that particular MW absorber can be reused multiple times. In this study, biochar as MW absorber was reused in whole experimentation for analyzing the weight loss of biochar, which may be owing to carbon residue reforming^33^.

The results showed uneven variations in biochar weight loss when MW power increased from 400 W, 700 W and 1000 W at different dosing and particle sizes. It was observed that as biochar dosing increased from 25 g, 37.5 g and 50 g, biochar weight loss first obtained maximum then decreased for 25 g and 37.5 g respectively whereas it further increased for 50 g of biochar dosing at particle sizes of 6 mm and 8 mm and all MW powers. However, the trend is reversed for particle size of 10 mm. Besides, minimum biochar weight loss occurred at 1.56% at 37.5 g and 400 W, and 1.87% at 50 g and 1000 W. As it observed higher/significant biochar weight loss happened as 2% to 10.4% due to higher heating rates which may help to increase syngas production in case of MW pyrolysis process. It means that higher biochar weight loss will contribute enhanced syngas because significantly less volatile and enriched carbon content is present in biochar. Microstructure of biochar may affect the MW heating, which needs further investigation. Since significant amount of porosity and higher surface area of absorber help to enhancing the heating rates under MW heating.

### Significant inferences of this study and future scope

Biochar has emerged and can be used as biofuel, soil conditioner, wastewater treatment, building materials, primary source for carbon-based nano-particles, energy storage material^34^ and electric rods in microbial fuel cells^35,36^. From the study analysis, biochar has great potential to use as MW absorber for performing MW torrefaction and MW pyrolysis owing to low cost, easy process for production and ease of availability. It observed that biochar has great potential to use as MW absorber for enhancing MW heating performance of torrefaction and pyrolysis processes. Conversely, it would help improve the quality of products produced from MW-based processes. However, this study is the first experimental work on analysis of heating performance of biochar under MW heating which needs to be investigated further analysis on different biochar, activated biochar as well as carbon/biochar-based catalysts.

## Conclusions

In present work, heating performance and microwave absorption efficiency of hardwood biochar under different biochar dosing, MW powers, and particle sizes were investigated. Findings exhibited that MW power and biochar dosing influenced the average heating rate significantly, means as MW power was increased as (400 W, 700 W and 1000 W), AHR also obtained higher as 17.5–65.4 °C/min whereas AHR first increased then no significant changes obtained as biochar dosing varied from 37.5 g to 50 g. AHR analyzed by employing the full factorial design which provided ~ 94.6% (R^2^) level of significance of this model. Model suggested that particle size of biochar influenced less on AHR. Besides, microwave absorption efficiency and biochar weight loss also investigated in which microwave absorption efficiency decreased as MW power increased means higher microwave absorption efficiency achieved at 400 W as compared to 700 W and 1000 W owing to low power and slow heating rates. However, it can be improved further when large amount of sample would be treating under MW heating. To examine the reusability of biochar, biochar weight loss was estimated by employing mass balance analysis after each experiment. It was testified that, overall, 2–10.4% of biochar weight loss occurred because of higher heating rates at higher powers and biochar dosing. This is silent advantage to increase the content syngas production in case of MW pyrolysis process. This loss could lead to the conversion of biochar into carbon monoxide because biochar is a carbon-based material, thereby potentially enhancing the composition of syngas during pyrolysis of any organic waste under microwave heating. The significant contribution of this study would promote low-cost biochar that can be employed as MW absorber to perform the MW-torrefaction and -pyrolysis instead of using expensive MW absorbers.

## Methods

### Materials and sampling

As discussed in the previous section, MW absorbers have significant role in achieving high temperatures under the MW heating for thermochemical processes. In this study, biochar is employed as MW absorber among all other absorbers produced from hardwood by conventional pyrolysis. The various properties of hardwood biochar have been tabulated in Table 2. It is assumed that MW penetration can affect the performance of hardwood biochar if it is reused again. In this work, the utilization of biochar was repeated to analyze the effect of MW penetration because different particle sizes and amounts of hardwood biochar were used. In this study, particle size and biochar dosing were chosen as (6 mm, 8 mm, 10 mm) and (25 g, 37.5 g, 50 g) depending upon the MW pyrolysis setup and 100 g of biomass sample. First, hardwood biochar blocks were crushed by using milling machine to reduce the particle size and then separate the biochar of different particle sizes with the help of different sieves. There may be some errors related to the particle size of hardwood biochar because the particle sizes > 7 mm and < 8 mm were assumed as 8 mm and others. Furthermore, the specific heat of hardwood biochar (C_p_) is varied in the range of ~ 1000–1200 J kg^−1^ K^−1^ which does not considerably change under higher temperatures as per literature which needs to be further analyzed^37^. Thus, C_p_ of hardwood biochar was assumed as 1200 J kg^−1^ K^−1^ for this study.

**Table 2:** Factorial experimental design of testing of biochar under MW heating.

Factor	Units	Low level (− 1)	Medium level (0)	High level (− 1)
Biochar dosing (X_1_)	G	25	37.5	50
Particle size (X_2_)	Mm	6	8	10
MW power (X_3_)	W	400	700	1000

### Experimental setup and design

**Figure 7:**
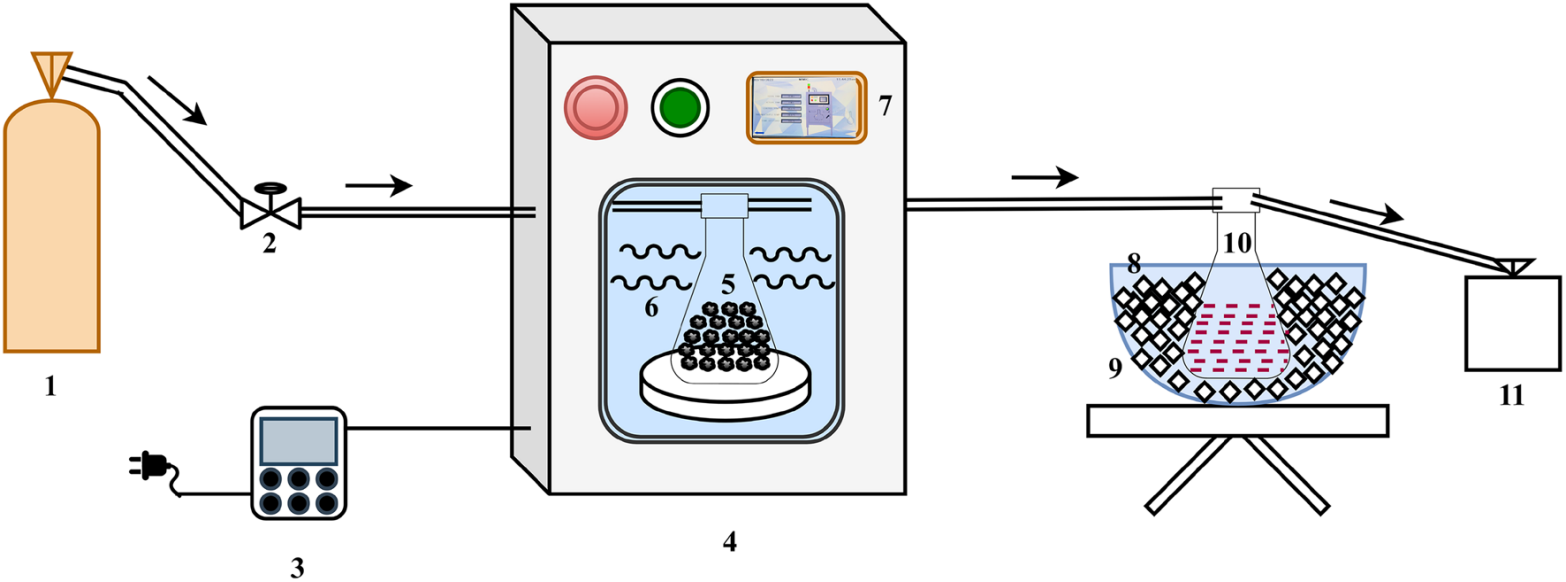
The experimental setup of MW heating in which the following accessories are attached such as (1) Inert gas cylinder (2) Regulator/Control valve (3) Energy meter for direct measurement (4) MW heating reactor (5) Quartz container for sample testing (6) MW irradiation from magnetron (7) Display for readings and control of setup (8) cooling container (9) Ice cubes (10) Collector of condensable gases (11) Tedlar bag.

MW heating system has been employed to test the samples for this study at the biofuel lab, Malaviya National Institute of Technology Jaipur, Rajasthan, India. Figure 7 depicts the systematic schematic of the experiment setup which consists of various components mainly the MW heating reactor, where the sample was kept in a quartz container which resists high-temperature shocks during heating. Moreover, the experiments were performed in an oxygen-free environment, which was created by flushing the inert gas from the gas cylinder, whereas nitrogen (N_2_) gas was used in this study.

Besides MW irradiation was produced from a magnetron of 2.45 GHz that provided the MW power of 1000 W and temperature rise was measured by temperature module which composed of high-precision K-type temperature sensors. Also, MW power and temperature range can be accustomed by using the control panel/display. During the experimentation, the samples of different particle sizes and dosing of biochar was filled in a quartz container. After that nitrogen was flushed for 15 min to remove the oxygen from the setup and then set the desired temperature of 600 °C which needed to achieve in this study within 10 min. The input power was examined by employing the energy measuring meter for each run. The setup was operated for 10 min and temperature rise was recorded for every 1 min for evaluation of average heating rate. As the temperature was set at 600 °C, the small amount of hardwood biochar can be reformed or decomposed, resulting in a loss in sample weight, which was also analyzed by weighing system.

### Factorial experimental design

For the analysis of the heating performance in terms of average heating rate (AHR) (°C/min), the experimental design was formulated using the full factorial experimental design of experiments in Design_Expert software. The factors affecting the AHR are tabulated in Table 2. Heating performance was evaluated from the AHR (°C/min) obtained from rise of temperature during each run in 10 min. These results are further employed to assess the microwave absorption efficiency in the next section. Table 2 shows several factors, such as MW power, biochar dosing, and particle size (W), that can affect the AHR (°C/min) as well as microwave absorption efficiency (%). To check the effect of these parameters on AHR (response) were varied as biochar dosing (25 g, 37.5 g, 50 g), particle size of biochar (6 mm, 8 mm, 10 mm), and MW power (400 W, 700 W and 1000 W). For preparing this experimental design, 3 levels and three factors full factorial design were employed to analyze the individual and joint effects of process parameters. Based on these parameters, the experimental matrix was formulated as possible combinations, tabulated in Table 3.

**Table 3:** Experimental matrix formulated from full factorial design.

Run	Factor 1	Factor 2	Factor 3	Response
	Biochar dosing (g)	Particle size (mm)	MW power (W)	Average heating rate (°C/min)
1	25	10	700	33.63
2	37.5	8	400	28.72
3	50	8	700	50.61
4	50	8	400	31.24
5	37.5	10	700	41.87
6	25	8	1000	56.54
7	37.5	8	700	47.58
8	37.5	6	700	42.13
9	25	6	400	18.94
10	50	10	400	32.24
11	37.5	10	1000	55.12
12	25	8	400	24.02
13	25	10	400	17.75
14	50	6	1000	60.03
15	37.5	8	1000	59.26
16	50	6	400	30.04
17	25	8	700	31.29
18	50	8	1000	54.56
19	50	10	700	45.8
20	37.5	6	1000	60.05
21	25	6	1000	58.08
22	50	6	700	41.6
23	25	6	700	37.05
24	25	10	1000	59.56
25	37.5	6	400	25.89
26	37.5	10	400	24.44
27	50	10	1000	65.4

### Theoretical analysis of data

As mentioned above, microwave absorption efficiency was evaluated by employing the standard formulas on experimental results obtained from the MW heating setup. Microwave absorption efficiency depends upon different types of factors, mainly input energy (E), energy absorbed by material (Q_1_), setup losses (Q_2_), and dissipation losses (Q_3_), which were evaluated as follows.

Input energy, E (kJ) is the electrical energy which was measured by direct measurement from digital energy meter. That input energy is converted into MW energy with the help of a magnetron, which is used for MW heating. There may be some magnetron losses, mainly heat loss as MW irradiation strikes the material and other heat loss related to high-frequency currents produced on the material’s surface during oscillation. These losses are considered the magnetron loss (Q_2_) in this study. Q_2_ typically may depend on the magnetron type and MW power. Further, there are some dissipation losses (Q_3_) due to MW heat losses, which is the ratio of the MW power absorbed by the material in the cavity to the MW power fed into the oven cavity. The losses in MW dissipation primarily consist of three components. The first component pertains to the energy that is reflected to the magnetron, contingent principally on the voltage standing wave ratio at the cavity's input end. The second component comprises energy loss within the cavity itself, primarily influenced by the cavity’s walls and the elements within it. The third component involves the MW energy escaping through the furnace outlet and exhaust hole^25^.

Besides the energy absorbed, Q_1_ (kJ) by hardwood biochar was evaluated by using Eq. (2).

2 $$Q_{1} = \smallint C_{p} m{\text{d}}T$$

where C_p_ is the specific heat (J kg/K), m (kg) is the mass of hardwood biochar, dT is the change in temperature (Final temperature − Initial Temperature (Considered room temperature for this study)).

Magnetron or MW irradiation device loss Q_2_ (kJ) can be evaluated using Eq. (3),

3 $${\text{Q}}_{2} = {\text{E}}{-}{\text{P}}*{\text{t}}$$

where E is the input energy (kJ) along with P is the MW power operated for desired time, t (mins).

MW dissipation loss (Q_3_) is the heat dissipated from the thermodynamic system to the surroundings and the container wall, which can be examined by Eq. (4).

4 $${\text{Q}}_{3} = {\text{E}}{-}{\text{Q}}_{1} {-}{\text{Q}}_{2}$$

MW absorption efficiency of biochar, ŋ is evaluated by employing the Eq. (5),

5 $$\eta = \frac{{Q_{1} }}{E} \times 100.$$

### Uncertainty analysis

It is necessary to examine the uncertainty analysis to reduce different types of errors related to instrumental, personal, and environmental errors. Among all errors, instrumental errors highly affect the results. In this study, uncertainty analysis was performed based on instrumental errors that can arise from different MW heating setup components, which are tabulated in Table 4. Equation (5) was employed to calculate the uncertainty as taken from 38. The overall uncertainty in research work was evaluated for all given uncertainties in Table 4, which obtained 2.31%, which can be considered as per literature.

$$\begin{aligned} {\text{Overall}}\;{\text{uncertainty}}: & \;{\text{Square}}\;{\text{root}}\;{\text{of}}\;{\text{data}}\;{\text{obtained}}\;{\text{from}}\;\left[ {\left( {{\text{Segregation}}\;{\text{of}}\;{\text{biochar}}\;{\text{as}}\;{\text{per}}\;{\text{size}}\;{\text{by}}\;{\text{Sievers}}} \right)^{2} } \right. \\ & \quad + \left( {{\text{Electronic}}\;{\text{weighing}}\;{\text{balance}}} \right)^{2} + \left( {{\text{Inert}}\;{\text{Gas}}\;{\text{Supply}}\;{\text{regulator}}} \right)^{2} + \left( {{\text{Flow}}\;{\text{meter}}} \right)^{2} \\ & \quad + \left( {{\text{Magnetron}}} \right)^{2} + \left( {{\text{Temperature}}\;{\text{sensors}}} \right)^{2} + \left( {{\text{Thermocouple}}\;{\text{values}}} \right)^{2} \\ & \quad \left. { + \left( {{\text{Container}}\;{\text{and}}\;{\text{sample}}\;{\text{surface}}\;{\text{temperature}}\;{\text{difference}}} \right)^{2} } \right] \\ & = 2.31\% \\ \end{aligned}$$

**Table 4:** Uncertainties of different instruments and their analysis used in this study.

Instrument name	Specifications	Average uncertainties (%)
Sievers	6, 8, 10 mm	1.2
Electronic weighing balance	0 to 120 g (< 0.001 g)	0.1
Inert Gas Supply regulator	Max. 4500 Psi	0.5
Flow meter	Max. 5000 Psi	0.5
Magnetron	0 to 1000 W	1.5
Temperature sensors	− 60 to 1200 °C	0.35
Thermocouple	0 to 900 °C	0.6
Container and sample surface temperature difference	1 to 4 °C	0.8

## Data Availability

The datasets used and/or analysed during the current study available from the corresponding author on reasonable request.
